# Association of genetic ancestry with outcomes and toxicity of idecabtagene vicleucel in patients with relapsed/refractory multiple myeloma

**DOI:** 10.1038/s41408-026-01489-9

**Published:** 2026-04-13

**Authors:** Christen M. Dillard, Hans Lee, Nilesh Kalariya, Naveen Subramanian, Oren Pasvolsky, Christopher Ferreri, Mahmoud Gaballa, Sheeba K. Thomas, Donna M. Weber, Melody Becnel, Gregory Kaufman, Muzaffar Qazilbash, Robert Z. Orlowski, Michelle A. T. Hildebrandt, Krina K. Patel

**Affiliations:** 1 Division of Cancer Medicine, The University of Texas MD Anderson Cancer Center, Houston, TX USA; 2 Department of Lymphoma & Myeloma, The University of Texas MD Anderson Cancer Center, Houston, TX USA; 3 UTHealth Houston McGovern Medical School, Houston, TX USA; 4 Department of Stem Cell Transplantation and Cellular Therapy, The University of Texas MD Anderson Cancer Center, Houston, TX USA; 5 Department of Experimental Therapeutics, The University of Texas MD Anderson Cancer Center, Houston, TX USA

**Keywords:** Myeloma, Translational research

## Introduction

Novel T-cell directed therapies, including chimeric antigen receptor (CAR)-T cells and bispecific antibodies, have transformed treatment for relapsed/refractory multiple myeloma (RRMM). Although myeloma remains largely incurable, BCMA-directed CAR-T therapy has demonstrated unprecedented efficacy with overall response rates (ORR) of 73–100% and complete response rates of 33–90% [1, 2]. However, racial and ethnic minorities, who experience disproportionate myeloma burden with higher incidence and mortality, remain underrepresented in clinical trials [3], leaving critical gaps in understanding safety and efficacy for these populations [4].

Post hoc analyses reveal inconsistencies. The KarMMa-3 subgroup analysis found comparable outcomes between Black and White patients [5], while real-world data from the US MM Immunotherapy Consortium revealed notable differences [6]. Black patients had higher levels of baseline inflammatory markers, increased cytokine release syndrome (CRS), and severe prolonged cytopenias, though progression free survival (PFS) or overall survival (OS) remained similar across groups.

These inconsistencies may reflect limitations of using race and ethnicity as analytical categories [7]. Because racial and ethnic identities are evolving and context-dependent, genetically inferred ancestry may provide a more precise measure of biologically relevant factors influencing CAR-T safety and efficacy. This study investigated associations between genetic ancestry and CAR-T outcomes.

## Methods

This single-center retrospective analysis included adults with RRMM who underwent apheresis for idecabtagene vicleucel (ide-cel) at MD Anderson Cancer Center (MDACC) (11/2018- 2/2023). Patients received treatment via clinical trial (KarMMa-2, KarMMa-3) or standard of care. Self-identified racial and ethnic categories included in the analysis were non-Hispanic White, non-Hispanic Black, and Hispanic/Latino patients with available biospecimens. This study was approved by the MDACC Institutional Review Board with waiver of informed consent.

Data were abstracted from electronic medical records, including demographics, disease characteristics, treatment details, and follow-up outcomes (response and adverse events). Organ involvement was defined as visceral extramedullary disease (EMD) involving major organs and/or myeloma renal disease, with bone-dependent and paraskeletal plasmacytomas excluded. High-risk disease features were defined a priori and are detailed in Table 1.

**Table 1:** Baseline patient characteristics by genetic ancestry.

	Overall (*N* = 49)	African Ancestry			European Ancestry			Admixed American Ancestry		
		≥75% (*N* = 9)	<75% (*N* = 40)	*P* value	≥50% (*N* = 30)	<50% (*N* = 19)	*P* value	≥30% (*N* = 8)	<30% (*N* = 41)	*P* value
**Race/Ethnicity**^***a***^ **(%)**										
Black	13 (26.5)	9 (100.0)	4 (10.0)	<0.001	0	13 (68.4)	<0.001	0	13 (31.7)	<0.001
White	26 (53.1)	0	26 (65.0)		25 (83.3)	1 (5.3)		0	26 (63.4)	
Hispanic/Latino	10 (20.4)	0	10 (25.0)		5 (16.7)	5 (26.3)		8 (100.0)	2 (4.9)	
**Male gender (%)**	29 (59.2)	4 (44.4)	25 (62.5)	0.32	19 (63.3)	10 (52.6)	0.46	6 (75.0)	23 (56.1)	0.32
**Age at diagnosis, median (IQR), y**	60.1 (51.5–66.6)	53.1 (48.7–65.0)	60.8 (53.3–67.0)	0.33	61.7 (53.5–67.3)	54.4 (46.2–65.0)	0.08	51.2 (41.1–60.8)	61.7 (53.1–67.6)	0.02
**Age at CAR T, median (IQR), y**	65.5 (58.7–73.3)	62.7 (61.8–73.3)	66.2 (58.0–72.8)	0.46	69.5 (59.4–75.9)	63.3 (53.3–71.6)	0.17	59.6 (51.0–65.5)	68.9 (60.1–73.4)	0.09
**ECOG PS** ≥ **2 (%)**	16 (32.6)	6 (66.7)	10 (25.0)	0.02	6 (20.0)	10 (52.6)	0.02	4 (50.0)	12 (29.3)	0.25
**R-ISS disease stage (%)**										
I	7 (17.1)	2 (22.2)	5 (15.6)	0.50	4 (16.7)	3 (17.7)	0.17	2 (33.3)	5 (14.3)	0.50
II	23 (56.1)	6 (66.7)	17 (53.1)		11 (45.8)	12 (70.6)		3 (50.0)	20 (57.1)	
III	11 (26.8)	1 (11.1)	10 (31.3)		9 (37.5)	2 (11.8)		1 (16.7)	10 (28.6)	
**High-risk disease features**^***b***^ **(%)**	38/42 (90.5)	9 (100.0)	29 (87.9)	0.27	20 (87.0)	18 (94.7)	0.39	6 (85.7)	32 (91.4)	0.64
Cytogenetics, high-risk^*c*^ (%)	27/45 (60.0)	7 (77.8)	20 (55.6)	0.22	13 (48.2)	14 (77.8)	0.047	3 (42.9)	24 (63.2)	0.31
BM plasma cells ≥50% (%)	10 (20.4)	5 (55.6)	5 (12.5)	0.004	3 (10.0)	7 (36.8)	0.02	3 (37.5)	7 (17.1)	0.19
Organ involvement^*d*^	17 (36.2)	6 (66.7)	11 (29.0)	0.03	7 (24.1)	10 (55.6)	0.03	3 (42.9)	14 (35.0)	0.25
**Prior therapy lines, median (IQR)**	6 (4–8)	7 (5–9)	5 (4–8)	0.32	5 (4–8)	7 (5–9)	0.03	6.5 (5–10)	5 (4–8)	0.20
**Prior ASCT (%)**	45 (91.8)	9 (100.0)	36 (90.0)	0.32	26 (86.7)	19 (100.0)	0.10	8 (100.0)	37 (90.2)	0.36
**Prior anti-BCMA therapy (%)**	10/37 (27.0)	1 (12.5)	9 (31.0)	0.30	6 (27.3)	4 (26.7)	0.97	2 (33.3)	8 (25.8)	0.70
**Triple class drug refractory (%)**	43 (87.8)	7 (77.8)	36 (90.0)	0.31	27 (90.0)	16 (84.2)	0.55	8 (100.0)	35 (85.4)	0.25
**Penta drug refractory**^***e***^ **(%)**	11 (23.4)	2 (22.2)	9 (23.7)	0.93	6 (21.4)	5 (26.3)	0.70	4 (50.0)	7 (18.0)	0.05
**Follow-up time, median (IQR), y**	17.0 (11.3–25.9)	18.9 (7.1–23.5)	15.7 (11.4–26.4)	0.88	16.4 (12.6–26.9)	17.8 (5.9–23.7)	0.33	13.2 (3.9–25.5)	17.4 (11.5–25.9)	0.60

^*a*^Race and ethnicity were reported by the patients and obtained from the electronic medical record.

^*b*^High-risk features include high risk cytogenetics, extramedullary disease, plasma cell leukemia, R-ISS stage 3, or response to bridging therapy < PR.

^*c*^High-risk cytogenetics include deletion 17p, t(4;14), t(14;16), gain or amplification of 1q.

^*d*^Organ involvement included visceral extramedullary disease and myeloma renal disease.

^*e*^Penta drug refractory refers to refractoriness to an anti-CD38 antibody, two proteasome inhibitors, and two immunomodulatory drugs.

*IQR* interquartile range, *y* years, *ECOG* Eastern Cooperative Oncology Group, *R-ISS* Revised-International Staging System, *BM* bone marrow, *ASCT* autologous stem cell transplant, *BCMA* B-cell maturation antigen.

Germline DNA was genotyped on the Illumina Human Genome Screening Array (v3.0) with imputation to TOPMed-r2. Continental genetic ancestry proportions (African [AFR], European [EUR], and Admixed American [AMR]) were estimated using ADMIXTURE by comparison to 1000 Genomes Project reference populations [8]. Patients were grouped by genetic ancestry using predefined cutoffs informed by the distribution of ancestry proportions within the cohort to generate analytically distinct groups independent of self-identified race/ethnicity: AFR ≥75% or < 75%, EUR ≥50% or <50%, AMR ≥30% or <30.

Efficacy outcomes included ORR, PFS, and OS, per International Myeloma Working Group criteria [9]. Safety assessments focused on CRS, immune effector cell-associated neurotoxicity syndrome (ICANS), and hematologic toxicities [10]. Data cutoff was 18 months post-infusion; survival data updated through 10/30/23.

Statistical analyses used chi-squared/Fisher’s exact tests for categorical variables and Kruskal-Wallis tests for continuous variables. Univariate and multivariable logistic regression models adjusted for cytogenetics, EMD, degree of pretreatment, and refractoriness. Kaplan–Meier methods estimated survival outcomes with log-rank testing. Two-sided *p* <0.05 was considered statistically significant.

## Results

### Patient Characteristics

Of 84 identified patients, 49 were eligible (26 White [53%], 13 Black [27%], and 10 Hispanic/Latino [20%]). Median age was 60.1 years at diagnosis and 65.5 years at CAR-T infusion. Most patients had ECOG scores of 0–1 (67%). The majority of patients (91%) had at least one high-risk feature, and 27 patients (60%) had high-risk cytogenetics. This heavily pretreated population received a median of 6 prior lines of therapy; 92% had prior autologous transplant and 27% had prior anti-BCMA therapy.

Self-identification aligned with genetic ancestry: all patients with ≥75% African ancestry (AFR) self-identified as Black, 83% with ≥50% European ancestry (EUR) self-identified as White, and all with ≥30% Admixed American ancestry (AMR) as Hispanic/Latino (Supplemental Fig. 1).

### Ancestry-Related Baseline Differences

Significant age differences emerged: patients with ≥50% EUR were older at diagnosis compared to those with <50% EUR (61.7 vs. 54.4), while those with predominant AMR were younger by over 10 years (*P* = 0.02).

Disease characteristics varied across ancestry groups. Patients with <50% EUR had higher rates of high-risk cytogenetics (*P* = 0.047). Those with ≥75% AFR demonstrated more aggressive disease features compared to those with <75% AFR: bone marrow plasma cell burden ≥50% (56% vs. 13%, *P* <0.01) and organ involvement including visceral EMD (67% vs. 29%, *P* = 0.03). Conversely, high EUR appeared protective with lower organ involvement (24% vs. 56%, *P* = 0.03) and plasmacytosis (10% vs. 37%, *P* = 0.02).

Treatment patterns differed: high EUR correlated with fewer prior lines of therapy (median 5 vs 7, *P* = 0.03), though triple-class and penta-drug refractory disease rates were similar. Performance status also varied significantly: patients with high AFR had ECOG ≥2 more frequently compared to those with low AFR (67% vs 25%; *P* = 0.02)(Supplemental Table 2).

Medication burden differed significantly (Supplemental Table 2) with higher AFR correlating with a greater number of medications versus low AFR (mean 9 vs. 8, *P* = 0.04), and fewer medications for higher EUR (mean 7 vs. 9, *P* = 0.04), potentially reflecting differences in comorbidity severity or management complexity. Higher AMR correlated with greater BMI (mean 35.6 vs. 27.8, *P* = 0.04), and there was a trend towards higher ferritin levels in patients with higher AFR.

### Efficacy Outcomes

At median 17-month follow-up, 42 of 48 evaluable patients achieved a measurable response. No significant differences in ORR, PFS or OS emerged by genetic ancestry in univariate and multivariable analyses, despite substantial baseline disparities (Supplemental Table 3).

### Safety Outcomes

CRS occurred in 86% of patients overall (2% grade ≥3)(Table 2). While all patients with high AFR developed CRS versus 80% of high EUR and 75% high AMR, differences were not statistically significant (*P* = 0.18 and *P* = 0.34, respectively).

**Table 2:** Toxicities by genetic ancestry.

	Overall (*N* = 49)	African Ancestry			European Ancestry			Admixed American Ancestry		
*N* (%)		≥75% (*N* = 9)	<75% (*N* = 40)	*P* value	≥50% (*N* = 30)	<50% (*N* = 19)	*P* value	≥30% (*N* = 8)	<30% (*N* = 41)	*P* value
**Cytokine release syndrome (CRS)**										
All grade	42 (85.7)	9 (100.0)	33 (82.5)	0.18	24 (80.0)	18 (94.7)	0.15	6 (75.0)	36 (87.8)	0.34
Grade ≥ 3	1 (2.0)	0	1 (2.5)	0.63	1 (33.3)	0	0.42	0	1 (2.4)	0.66
**Neurotoxicity/Immune effector cell-associated neurotoxicity syndrome (ICANS)**										
All grade	7 (14.3)	3 (33.3)	4 (10.0)	0.07	4 (13.3)	3 (15.8)	0.81	0	7 (17.1)	0.21
Grade ≥ 3	2 (4.1)	1 (11.1)	1 (2.5)	0.24	1 (3.3)	1 (5.3)	0.74	0	2 (4.9)	0.52
**CRS/ICANS interventions**										
Tocilizumab	32 (65.3)	6 (66.7)	26 (65.0)	0.92	20 (66.7)	12 (63.2)	0.80	5 (62.5)	27 (65.9)	0.86
Steroids	5 (10.2)	2 (22.2)	3 (7.5)	0.19	3 (10.0)	2 (10.5)	0.95	0	5 (12.2)	0.30
Anakinra	1 (2.0)	1 (11.1)	0	0.03	0	1 (5.3)	0.20	0	1 (2.4)	0.66
**Anemia (Grade** ≥ **3)**										
30 days	4/49 (8.2)	1 (11.1)	3 (7.5)	0.72	2 (6.7)	2 (10.5)	0.63	2 (25.0)	2 (4.9)	0.057
60 days	3/41 (7.3)	0	3 (8.8)	0.41	2 (8.0)	1 (6.3)	0.83	0	3 (8.8)	0.41
90 days	2/42 (4.8)	0	2 (5.9)	0.48	1 (3.7)	1 (6.7)	0.67	1 (14.3)	1 (2.9)	0.20
**Neutropenia (Grade** ≥ **3)**										
30 days	12/48 25.0)	4 (50.0)	8 (20.0)	0.07	6 (20.0)	6 (33.3)	0.30	1 (12.5)	11 (27.5)	0.37
60 days	8/41 19.5)	1 (14.3)	7 (20.6)	0.70	5 (20.0)	3 (18.8)	0.92	1 (14.3)	7 (20.6)	0.70
90 days	9/42 (21.4)	3 (37.5)	6 (17.7)	0.22	2 (7.4)	7 (46.7)	0.003	2 (28.6)	7 (20.0)	0.61
**Thrombocytopenia (Grade** ≥ **3)**										
30 days	23/49 (46.9)	6 (66.7)	17 (42.5)	0.19	13 (43.3)	10 (52.6)	0.53	4 (50.0)	19 (46.3)	0.85
60 days	12/41 (29.3)	3 (42.9)	9 (26.5)	0.39	5 (20.0)	7 (43.8)	0.10	2 (28.6)	10 (29.4)	0.97
90 days	11/42 (26.2)	3 (37.5)	8 (23.5)	0.42	5 (18.5)	6 (40.0)	0.13	2 (28.6)	9 (25.7)	0.88
**Infectious complications**										
Any infection	20 (40.8)	7 (77.8)	13 (32.5)	0.01	9 (30.0)	11 (57.9)	0.053	2 (25.0)	18 (43.9)	0.32
Bacterial	5/20 (25.0)	1 (14.3)	4 (30.8)	0.42	4 (44.4)	1 (9.1)	0.07	0	5 (27.8)	0.39
Viral	14/20 (70.0)	6 (85.7)	8 (61.5)	0.26	5 (55.6)	9 (81.8)	0.20	2 (100.0)	12 (66.7)	0.33
Severe infection	3/18 (16.7)	1 (16.7)	2 (16.7)	1.00	1 (12.5)	2 (20.0)	0.67	1 (50.0)	2 (12.5)	0.18

Neurotoxic events (ICANS) affected 14% overall (4% grade ≥3). Patients with high AFR had numerically higher rates of ICANS compared to those with low AFR (33% vs. 10%), though not statistically significant.

Grade ≥3 neutropenia at 90 days was significantly more common with low EUR versus high EUR (47% vs. 7%, *P* = 0.003). High AFR showed numerically higher rates compared to low AFR at 30 days (50% vs. 20%, *P* = 0.07) and 90 days (38% vs. 18%, *P* = 0.22).

Infectious complications occurred in 41% overall and were significantly more frequent with higher AFR (78% vs. 33%, *P* = 0.01). The majority of infections were viral (70%), and 17% of patients developed severe infections requiring intensive care.

## Discussion

This study demonstrates equitable efficacy with anti-BCMA CAR-T cell therapy across diverse genetic ancestry groups despite significant baseline disparities. Patients with higher proportions of AFR and AMR ancestry, presenting with worse baseline health status and more heavily pretreated disease, achieved similar response and survival to those with greater EUR ancestry. However, important safety differences emerged, particularly regarding infections and hematologic toxicity.

Patients with high EUR were healthier at baseline–fewer medications, better performance status, fewer prior therapies–while high AFR correlated with poorer functional status, higher medication burden, and more pretreatment. Despite these substantial baseline differences, efficacy outcomes remained remarkably consistent across ancestry groups.

In contrast to efficacy outcomes, toxicity profiles varied significantly by ancestry. Patients with high AFR experienced more infections and prolonged neutropenia. Critically, most infections were viral rather than bacterial, indicating T-cell-mediated immunologic susceptibility beyond neutropenia alone. This pattern aligns with prior anti-BCMA CAR-T studies and suggests differences in immune recovery [11].

Several mechanisms may explain these findings. Genetic variants affecting immune response vary across populations and may influence post-CAR-T outcomes [12]. Ancestry-related variation in drug metabolism could affect lymphodepletion exposure and bone marrow recovery. Although our sample size limits definitive conclusions, we observed trends toward higher baseline ferritin levels and CRS rates in patients with AFR, suggesting heightened inflammatory responses. Delayed hematologic recovery and elevated ferritin predict severe infection in CAR-T recipients [13, 14].

Our results are consistent with emerging data showing CAR-T mitigates adverse prognostic factors in myeloma [15]. Studies of ide-cel demonstrate no differences in PFS or OS by race/ethnicity, even among patients with higher inflammatory markers, and meta-analytic data suggest CAR-T elicits deep, durable responses across high-risk subgroups. These findings have immediate implications: patients with AFR ancestry may benefit from enhanced infection monitoring and prophylaxis, while consistent efficacy across ancestry groups supports broader access initiatives.

Study strengths include use of genetically inferred ancestry data, which more directly captures inherited biological variation than self-reported race or ethnicity, along with a racially and ethnically diverse cohort with detailed follow-up. Limitations include the single-institution, retrospective design, a small sample size, and reliance on 1000 Genomes reference populations, which may not fully represent all sources of genetic diversity. Genetically inferred ancestry reflects similarity to continental reference populations rather than social categories, and does not account for social, environmental, or structural factors that also influence outcomes.

Future research should examine immune reconstitution, lymphocyte recovery, and hypogammaglobulinemia across ancestry groups. Increasing clinical trial diversity remains essential for prospective analyses. Notably, patients in this cohort with higher AFR were disproportionately likely to have been ineligible for key CAR-T trials, highlighting systemic barriers requiring attention.

CAR-T achieves equitable efficacy across ancestry groups despite baseline disparities, offering hope for reducing traditional disparities in myeloma outcomes. However, safety profile differences warrant consideration for risk stratification and supportive care. Future research incorporating both genetic and social determinants will be essential to optimize CAR-T outcomes while addressing systemic healthcare barriers.

## Supplementary information

Supplemental table clean

## Data Availability

Data from this study are not publicly available to maintain patient data privacy. Requests for de-identified data can be made to MATH or KKP and provided upon reasonable request.
