# Supplementary material for: Association of genetic ancestry with outcomes and toxicity of idecabtagene vicleucel in patients with relapsed/refractory multiple myeloma
Source: Blood Cancer J. 2026 Apr 13;16(1):59. doi: 10.1038/s41408-026-01489-9 (PMC13077055; doi:10.1038/s41408-026-01489-9)

**Supplementary Tables & Figures**

| **Supplementary Table 1. Patient characteristics by self-identified race/ethnicity** | | | | |
| --- | --- | --- | --- | --- |
|  | **OVERALL** | **White** | **Black** | **Hispanic/Latino** |
| **Number of Patients (N, %)** | 49 | 26 (53.0) | 13 (26.5) | 10 (20.4) |
| **Gender (N, %)** |  |  |  |  |
| Female | 20 (40.8) | 8 (30.8) | 8 (61.5) | 4 (40.0) |
| Male | 29 (59.2) | 18 (69.2) | 5 (38.5) | 6 (60.0) |
| **Age at Diagnosis, years (N, %)** |  |  |  |  |
| < 65 years | 34 (69.4) | 16 (61.5) | 9 (69.2) | 9 (90.0) |
| ≥ 65 years | 15 (30.6) | 10 (38.5) | 4 (30.8) | 1 (10.0) |
| Median (IQR) | 60.1 (51.5-66.6) | 61.8 (56.8-69.8) | 58.6 (48.7-67.6) | 51.0 (40.2-60.1) |
| **Median Age at CAR T, years (IQR)** | 65.5 (58.7-73.3) | 70.2 (60.1-77.4) | 63.3 (61.8-73.3) | 59.0 (48.7-65.5) |
| **ECOG PS at CAR T referral (N, %)** |  |  |  |  |
| 0-1 | 33 (67.4) | 22 (84.6) | 5 (38.5) | 6 (60.0) |
| ≥ 2 | 16 (32.6) | 4 (15.4) | 8 (61.5) | 4 (40.0) |
| **R-ISS stage at referral (N, %)** |  |  |  |  |
| I | 7 (14.3) | 3 (11.5) | 2 (15.4) | 2 (20.0) |
| II | 15 (30.6) | 7 (26.9) | 4 (30.8) | 4 (40.0) |
| III | 6 (12.2) | 3 (11.5) | 2 (15.4) | 1 (10.0) |
| Unknown | 21 (42.9) | 13 (50.0) | 5 (38.5) | 3 (30.0) |
| **Disease Characteristics (N, %)** |  |  |  |  |
| Any High-Risk Features | 38/42 (90.5) | 18/21 (85.7) | 13/13 (100.0) | 7/8 (87.5) |
| High-Risk Cytogenetics | 27/45 (60.0) | 14/24 (58.3) | 10/12 (83.3) | 3/9 (33.3) |
| del(17p) | 13/44 (29.6) | 8/23 (34.8) | 2/12 (16.7) | 3/9 (33.3) |
| t(4;14) | 4/44 (10.8) | 3/23 (13.0) | 0/12 | 1/9 (8.3) |
| t(14;16) | 1/43 (2.7) | 1/23 (4.4) | 0/12 | 0/8 |
| gain/amp 1q | 22/48 (45.8) | 13/26 (50.0) | 7/12 (58.3) | 2/10 (20.0) |
| High Bone Marrow Plasma Cell Burden | 10/49 (20.4) | 2 (7.7) | 5 (38.5) | 3 (30.0) |
| Extramedullary Disease | 15/47 (31.9) | 6 (25.0) | 5 (38.5) | 4 (40.0) |
| Organ Involvement | 17/47 (36.2) | 6 (24.0) | 8 (61.5) | 3 (33.3) |
| **Myeloma Subtype (N, %)** |  |  |  |  |
| Intact immunoglobulin | 35 (72.9) | 17 (68.0) | 10 (76.9) | 8 (80.0) |
| Light chain | 9 (18.8) | 5 (20.0) | 3 (23.1) | 1 (10.0) |
| Oligo-/nonsecretory | 4 (8.3) | 3 (12.0) | 0 | 1 (10.0) |
| **Prior Therapies (N, %)** |  |  |  |  |
| Median prior therapies (range) | 6 (4-8) | 5 (3-7) | 8 (4.5-10) | 6.5 (5-10) |
| Number of prior therapies ≥ 4 | 41 (83.7) | 19 (73.1) | 12 (92.3) | 10 (100.0) |
| Prior autologous SCT | 45 (91.8) | 22 (84.6) | 13 (100.0) | 10 (100.0) |
| Prior allogeneic SCT | 0/36 | 0 | 0 | 0 |
| Prior anti-BCMA therapy | 9/36 (25.0) | 4 (23.5) | 3 (27.3) | 2 (25.0) |
| **Refractory Status (N, %)** |  |  |  |  |
| Triple Class Refractory | 43 (87.8) | 23 (88.5) | 10 (76.9) | 10 (100.0) |
| Penta Drug Refractory | 11 (23.4) | 3 (12.5) | 3 (23.1) | 5 (50.0) |
| **Median Follow-up Time (months, IQR)** | 17.0 (11.3-25.9) | 17.2 (12.6-26.9) | 17.0 (6.1-19.0) | 13.0 (4.4-23.7) |
| **Genetic Ancestry (median %, IQR)** |  |  |  |  |
| AFR | 3.44 (1.9-64.8) | 2.0 (1.6-3.3) | 79.0 (71.9-85.1) | 3.4 (3.1-5.9) |
| EUR | 82.3 (22.2-94.2) | 94.1 (91.2-95.0) | 18.2 (10.0-22.2) | 46.3 (15.9-68.0) |
| AMR | 1.3 (0.1-3.9) | 1.0 (0-1.8) | 1.2 (0.6-2.7) | 47.0 (28.1-73.2) |

**Supplementary Table 2. Clinical characteristics at lymphodepletion and/or CAR-T infusion by genetic ancestry**

|  | **Overall  (N = 49)** | **African Ancestry** | |  | | | | | **European Ancestry** | | | | | |  | | | **American Ancestry** | | | | | | |  | | | |  |  |  |
| --- | --- | --- | --- | --- | --- | --- | --- | --- | --- | --- | --- | --- | --- | --- | --- | --- | --- | --- | --- | --- | --- | --- | --- | --- | --- | --- | --- | --- | --- | --- | --- |
|  |  | **≥ 75% (N=9)** | **< 75% (N=40)** | | ***P* value** | | | **≥ 50% (N=30)** | | | | | **< 50% (N=19)** | | | ***P* value** | | | **≥ 30% (N=8)** | | | | **< 30% (N=41)** | | | ***P* value** | | | |  |  |
| **Comorbidities, mean (StdDev)** |  |  |  | |  | |  | | |  | | | |  | | | | |  | | | |  |  | | | | | |  |  |
| HCl-HT score^†^ | 3 (1-5) | 2 (2-5) | 3 (1-5) | | 0.94 | 2 (1-5) | | | | | | 3 (2-5) | | | | | 0.29 | | 2.5 (2-4) | | | 3 (1-5) | | | | | 0.79 | | |  |  |
| No. of medications | 8 (5-12) | 9 (7-17) | 8 (4.5-11) | | 0.04 | 7 (4-10) | | | | | | 9 (6-15) | | | | | 0.04 | | 8 (3.5-11.5) | | | 8 (5-12) | | | | | 0.60 | | |  |  |
| BMI | 28.1  (25.0-32.2) | 27.8  (24.0-30) | 28.2  (25.0-32.4) | | 0.80 | 26.9  (24.8-30.4) | | | | | | 30  (26.7-35.6) | | | | | 0.16 | | 35.6  (26.0-41.3) | | | 27.8  (24.8-30.7) | | | | | 0.04 | | |  |  |
| **Labs, mean (StdDev)** |  |  |  | |  |  | | | | |  | | | | | |  | | |  |  | | | | | | |  | | |  |
| Ferritin, ng/mL | 428  (134-1044) | 1368  (227-2823) | 336  (125-717) | | 0.06 | 236  (128-668) | | | | | 523  (201-2692) | | | | | | 0.12 | | | 784  (183-1230) | 370  (134-766) | | | | | | | 0.43 | | |  |
| CRP, mg/L | 8.8  (3.8-16.3) | 16.1  (6.4-35.4) | 8.4  (3.6-15.6) | | 0.14 | 7.3  (3.4-15.8) | | | | | 9.6  (6.4-33.2) | | | | | | 0.12 | | | 10.7  (4.6-49.9) | 8.5  (3.8-16.3) | | | | | | | 0.59 | | |  |
| CrCl, mL/min | 76.6  (51.2-103.6) | 69.5  (50.0-134.8) | 80.9  (53.0-101.0) | | 0.82 | 78.7  (49.2-95.9) | | | | | 76.3  (63.5-146.0) | | | | | | 0.24 | | | 95.0  (81.7-133.4) | 75.7  (50.0-98.5) | | | | | | | 0.15 | | |  |
| eGFR, mL/min/1.73m^2^ | 75 (62-90) | 90 (52-96) | 75 (62-88) | | 0.56 | 75 (59-83) | | | | | 90 (62-103) | | | | | | 0.04 | | | 94 (75-102) | 74 (59-88) | | | | | | | 0.24 | | |  |
| **Response to bridging (%)** |  |  |  | |  |  | | | | |  | | | | | |  | | |  |  | | | | | | |  | | |  |
| PR or better | 3/42 (7.1) | 0 | 3 (9.1) | |  | 1 (4.0) | | | | | 2 (11.8) | | | | | |  | | | 2 (25.0) | 1 (2.9) | | | | | | |  | | |  |
| SD/PD | 38/42 (90.5) | 9 (100.0) | 29 (87.9) | | 0.55 | 23 (92.0) | | | | | 15 (88.2) | | | | | | 0.46 | | | 6 (75.0) | 32 (94.1) | | | | | | | 0.09 | | |  |
| Unknown | 1/42 | 0 | 1 (3.0) | |  | 1 (4.0) | | | | | 0 | | | | | |  | | | 0 | 1 (2.9) | | | | | | |  | | |  |
| **Fludarabine dose reduction (%)** | 12/47 (25.5) | 4 (44.4) | 8 (21.1) | | 0.15 | 6 (20.7) | | | | | 6 (33.3) | | | | | | 0.33 | | | 1 (14.3) | | 11 (27.5) | | | | | | 0.46 | | |  |
| **KarMMa trial exclusion criteria (%)** | | | | |  |  | | | | |  | | | | | |  | | |  | |  | | | | | |  | | |  |
| Meeting exclusion criteria, overall | 26 (53.1) | 6 (66.7) | 20 (50.0) | | 0.37 | 16 (53.3) | | | | | 10 (52.6) | | | | | | 0.96 | | | 3 (37.5) | | 23 (56.1) | | | | | | 0.33 | | |  |
| Organ dysfunction^§^ | 14 (28.6) | 5 (55.6) | 9 (22.5) | | 0.047 | 8 (26.7) | | | | | 6 (31.6) | | | | | | 0.71 | | | 1 (12.5) | | 13 (31.7) | | | | | | 0.27 | | |  |
| Cytopenias | 6 (12.2) | 3 (33.3) | 3 (7.5) | | 0.03 | 3 (10.0) | | | | | 3 (15.8) | | | | | | 0.55 | | | 0 | | 6 (14.6) | | | | | | 0.25 | | |  |
| Performance Status | 6 (12.2) | 1 (11.1) | 5 (12.5) | | 0.91 | 3 (10.0) | | | | | 3 (15.8) | | | | | | 0.55 | | | 1 (12.5) | | 5 (12.5) | | | | | | 0.98 | | |  |
| † Denotes a modified Hematopoietic Cell Transplantation Comorbidity Index (HCT-CI) score, which included eGFR <60 as a criterion.  § Organ dysfunction includes renal, cardiopulmonary, and hepatic disease. *Abbreviations—PR: partial response, SD/PD: stable disease/progressive disease, StdDev: standard deviation, CRP: C-reactive protein, CrCl: creatinine clearance, eGFR: estimated glomerular filtration rate, BMI: body mass index* | | | | | | | | | | | | | | | | | | | | | | | | | | | | | | | |

**Supplementary Table 3. Survival outcomes by genetic ancestry**

|  | **Overall  (N = 49)** | | **African Ancestry** | |  | | | **European Ancestry** | |  | | | **American Ancestry** | |  | |
| --- | --- | --- | --- | --- | --- | --- | --- | --- | --- | --- | --- | --- | --- | --- | --- | --- |
|  |  |  | **≥ 75% (N=9)** | **< 75% (N=40)** | | ***P* value** | | **≥ 50% (N=30)** | **< 50% (N=19)** | | ***P* value** | | **≥ 30% (N=8)** | **< 30% (N=41)** | | ***P* value** |
| **Overall Response (N=48)** | | | |  | |  | |  |  | |  | |  |  | |  |
| Response | 42 (87.5) | | 8 (88.9) | 34 (87.2) | | 0.89 | | 25 (86.2) | 17 (89.5) | | 0.74 | | 6 (85.7) | 36 (87.8) | | 0.88 |
| No response | 6 (12.5) | | 1 (11.1) | 5 (12.8) | |  | | 4 (13.8) | 2 (10.5) | |  | | 1 (14.3) | 5 (12.2) | |  |
| Multivariable OR (95% CI)^a^ | | 2.09 (0.08-53.21) | | Reference | | 0.66 | 0.90 (0.05-17.63) | | Reference | | 0.95 | 0.08 (0.00-1.78) | | Reference | | 0.11 |
| **PFS at 3 months (N=49)** | | |  |  | |  | |  |  | |  | |  |  | |  |
| No progression | 39 (79.6) | | 8 (88.9) | 31 (77.5) | | 0.44 | | 25 (83.3) | 14 (73.7) | | 0.41 | | 5 (62.5) | 34 (82.9) | | 0.19 |
| Progression | 10 (20.4) | | 1 (11.1) | 9 (22.5) | |  | | 5 (16.7) | 5 (26.3) | |  | | 3 (37.5) | 7 (17.1) | |  |
| Multivariable OR (95% CI)^b^ | | 4.45 (0.35-56.35) | | Reference | | 0.25 | 1.30 (0.19-8.99) | | Reference | | 0.79 | 0.46 (0.04-4.97) | | Reference | | 0.53 |
| **PFS at 6 months (N=48)** | | |  |  | |  | |  |  | |  | |  |  | |  |
| No progression | 33 (68.8) | | 7 (77.8) | 26 (66.7) | | 0.52 | | 21 (70.0) | 12 (66.7) | | 0.81 | | 4 (57.1) | 29 (70.7) | | 0.47 |
| Progression | 15 (31.3) | | 2 (22.2) | 13 (33.3) | |  | | 9 (30.0) | 6 (33.3) | |  | | 3 (42.9) | 12 (29.3) | |  |
| Multivariable OR (95% CI)^b^ | | 2.78 (0.41-18.60) | | Reference | | 0.29 | 0.60 (0.12-3.01) | | Reference | | 0.54 | 1.72 (0.21-14.05) | | Reference | | 0.61 |
| **OS at 6 months (N=49)** | | |  |  | |  | |  |  | |  | |  |  | |  |
| Alive | 45 (91.8) | | 8 (88.9) | 37 (92.5) | | 0.72 | | 29 (96.7) | 16 (84.2) | | 0.12 | | 6 (75.0) | 39 (95.1) | | 0.06 |
| Deceased | 4 (8.2) | | 1 (11.1) | 3 (7.5) | |  | | 1 (3.3) | 3 (15.8) | |  | | 2 (25.0) | 2 (4.9) | |  |
| Multivariable OR (95% CI)^c^ | | 1.65 (0.11-25.23) | | Reference | | 0.72 | | N/A | N/A | | N/A | | N/A | N/A | | N/A |

**Abbreviations:** OR, odds ratio; CI, confidence interval; PFS, progression-free survival; OS, overall survival

**Footnotes:**

ᵃ Multivariable OR adjusted for high-risk cytogenetics, extramedullary disease, number of prior lines of treatment, and triple-class refractory status. Models predict no response; OR > 1 indicates higher odds of no response. Analysis N=42 due to missing covariates.

ᵇ Multivariable OR adjusted for high-risk cytogenetics, extramedullary disease, number of prior lines of treatment, and triple-class refractory status. Models predict progression; OR < 1 indicates higher odds of progression (lower odds of remaining progression-free). Analysis N=42 due to missing covariates.

ᶜ Multivariable OR adjusted for extramedullary disease and number of prior lines of treatment. High-risk cytogenetics and triple-class refractory status were omitted due to perfect prediction. Models predict death; OR > 1 indicates higher odds of death. Analysis N=23 after listwise deletion for African ancestry analysis. European and American ancestry models could not be estimated due to perfect prediction and numerical instability, respectively.

**
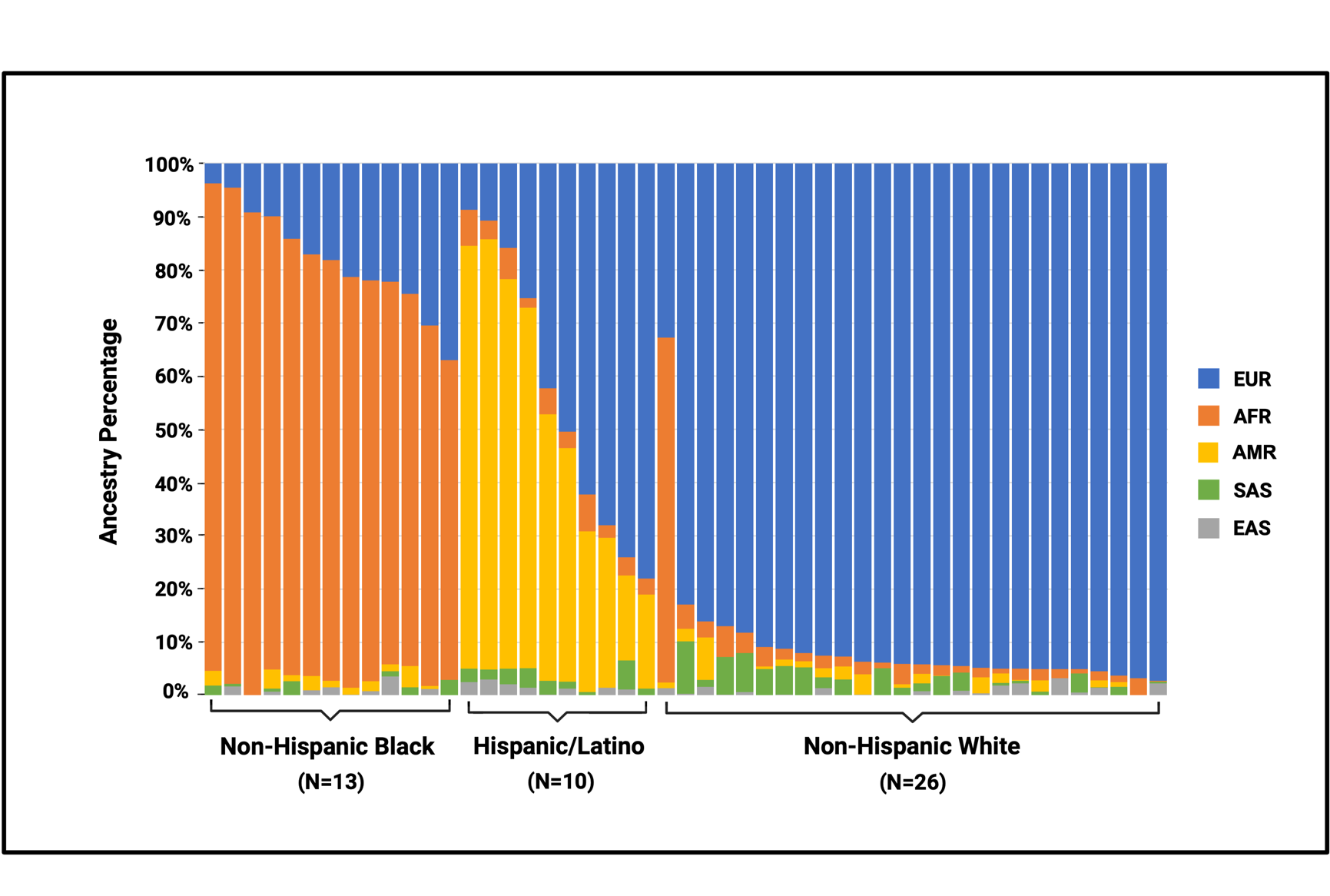
Supplementary Figure 1.** Genetic ancestry of patient population by self-identified race/ethnicity. Abbreviations: EUR = European, AFR = African, AMR = American, SAS = South Asian, EAS = East Asian.

**Supplementary Figure 2.** Overall response rate (ORR) to treatment by genetic ancestry. Of 48 evaluable patients, 42 had a measurable response to treatment.


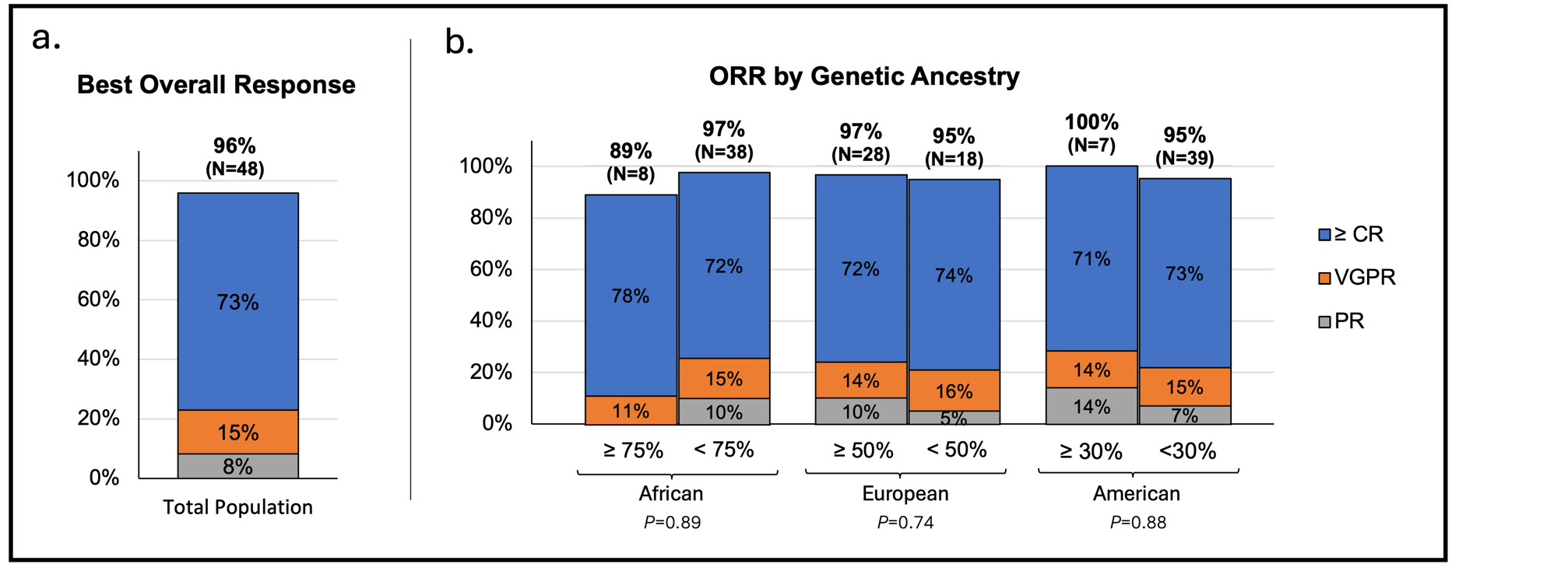

Supplement: Supplementary file 1 — Supplemental table clean [file 41408_2026_1489_MOESM1_ESM.docx]
